# Supplementary material for: Serum Glycoprotein Markers in Nonalcoholic Steatohepatitis and Hepatocellular Carcinoma
Source: J Proteome Res. 2022 Mar 14;21(4):1083–94. doi: 10.1021/acs.jproteome.1c00965 (PMC8981307; doi:10.1021/acs.jproteome.1c00965)
Supplement: Supplementary file 1 — pr1c00965_si_001.pdf [file pr1c00965_si_001.pdf]

Serum glycoprotein markers in non-alcoholic steatohepatitis and hepatocellular carcinoma

Prasanna Ramachandran, Gege Xu, Hector H. Huang, Rachel Rice, Bo Zhou, Klaus Lindpaintner and Daniel Serie*

InterVenn Biosciences, South San Francisco, CA 94080

Supporting information

Figure S1. Representative example for chromatographic separation of different glycoforms of the glycopeptide - VVLHPN*YSQVDIGLIK from haptoglobin

Figure S2. Principal component analysis of serum from control, NASH and HCC subjects using potential "progression markers". The X-axis represents the first principal component and Y-axis represents the second principal component. Each dot represents first and second principal component coordinates of a subject

Figure S3. Relative abundance of common glycoforms *5400, 5401*, *5411*, *5412* in control, NASH and HCC serum across all 73 glycoproteins studied. Columns indicate cumulative relative abundances of glycans among the glycoproteins being monitored

Figure S4. Relative abundances of common glycoforms *6501*, *6511*, *6512*, *6502*, *6512*, *6503*, *6513* in control, NASH, and HCC serum across all 73 glycoproteins studies. Columns indicate cumulative relative abundances of glycans among the glycoproteins being monitored

Figure S5. Relative abundances of common glycoforms *7600*, *7602, 7604*, *7613*, *7614* in control, NASH and HCC serum across all 73 glycoproteins studied. Columns indicate cumulative relative abundances of glycans among the glycoproteins being monitored

Figure S6. Normalized abundances of peptide and glycopeptides of AGP1 in control, NASH, and HCC serum across all 73 glycoproteins studied. Columns indicate normalized abundances of a certain type of glycans

Figure S7. Normalized abundances of peptide and glycopeptides of HPT in control-, NASH- and HCC-samples. Columns indicate normalized abundances of a certain type of glycans

Figure S8. Normalized abundances of peptide and glycopeptides of CFAH in control-, NASH- and HCC-samples. Columns indicate normalized abundances of glycans

Figure S9. Normalized abundance of peptide and glycopeptides of A1AT in control-, NASH- and HCC-samples. Columns indicate normalized abundances of glycans

Figure S10. ROC curves generated using LOOCV in validation training and test sets, as well as applied to HCC and control samples in the discovery set

Figure S11. (a) Network of fucosyltransferases and target glycoproteins. Solid lines represent a direct interaction between molecules. Dotted lines represent an indirect interaction. (b) Network of sialyltransferases and target glycoproteins

Table S1. Summary of NASH patients in the discovery dataset

Table S2. Summary of HCC patients in the discovery dataset

Table S3. Multiplicative differences, Student's t-test p-values, and FDR values for unidirectionally differentially expressed glycopeptides ("progression markers")

Table S4. Multiplicative difference between NASH/control and HCC/control

Table S5. Glycan code and structure

Table S6. IPA analysis - top ten upstream regulators

Fig S1.

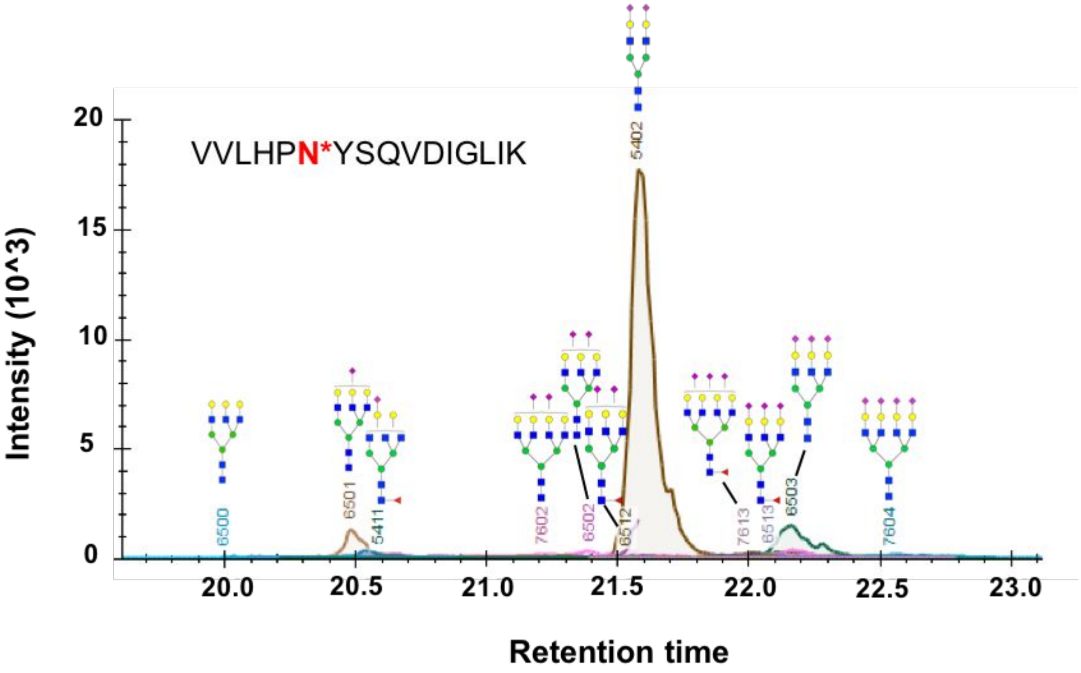

VVLHPN*YSQVDIGLIK
Intensity (10^3)
Retention time
20
15
10
5
0
20.0
20.5
21.0
21.5
22.0
22.5
23.0
6500
6501
5411
7602
6502
6512
5402
7613
6513
6503
7604

Figure S2.

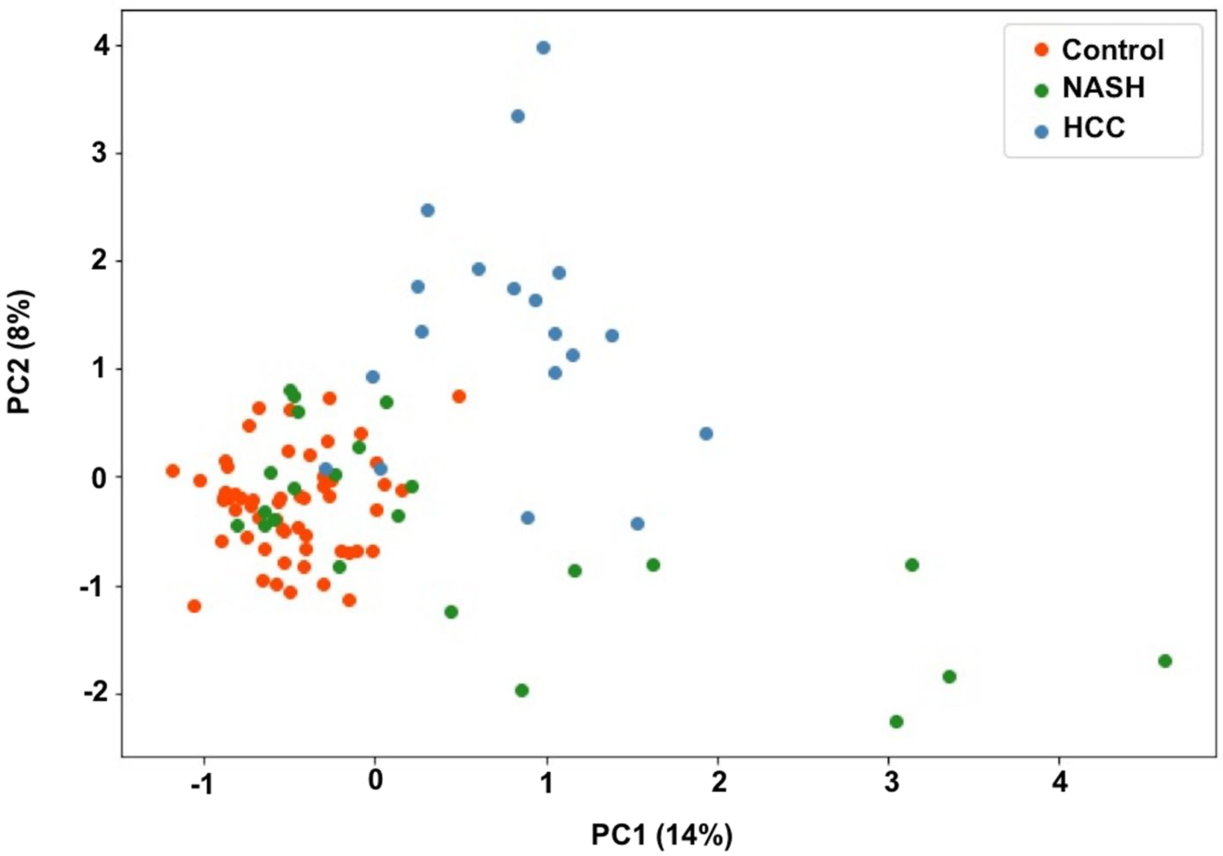
Control
NASH
HCC
PC2 (8%)
PC1 (14%)
4
3
2
1
0
-1
-2
-1
0
1
2
3
4

Figure S3.

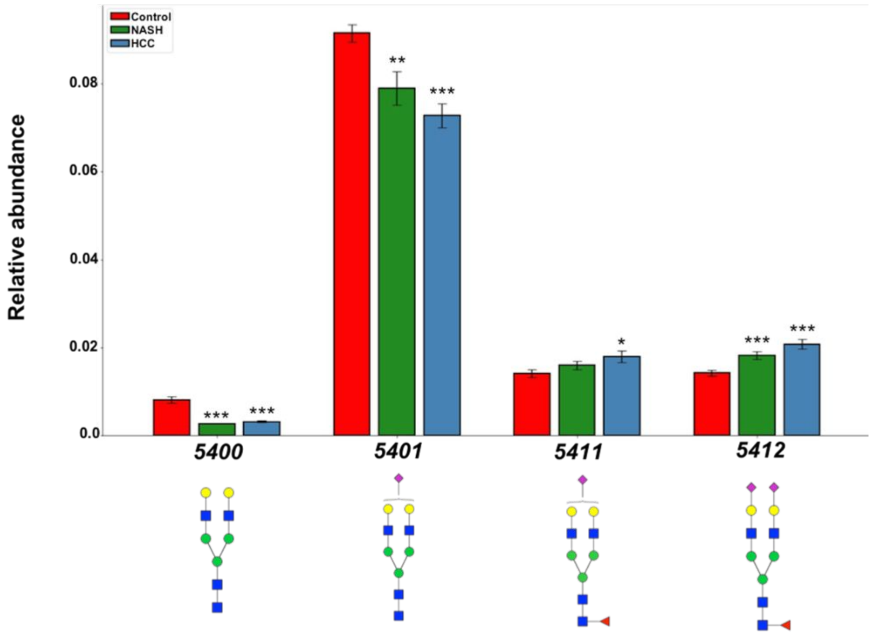

Figure S4.

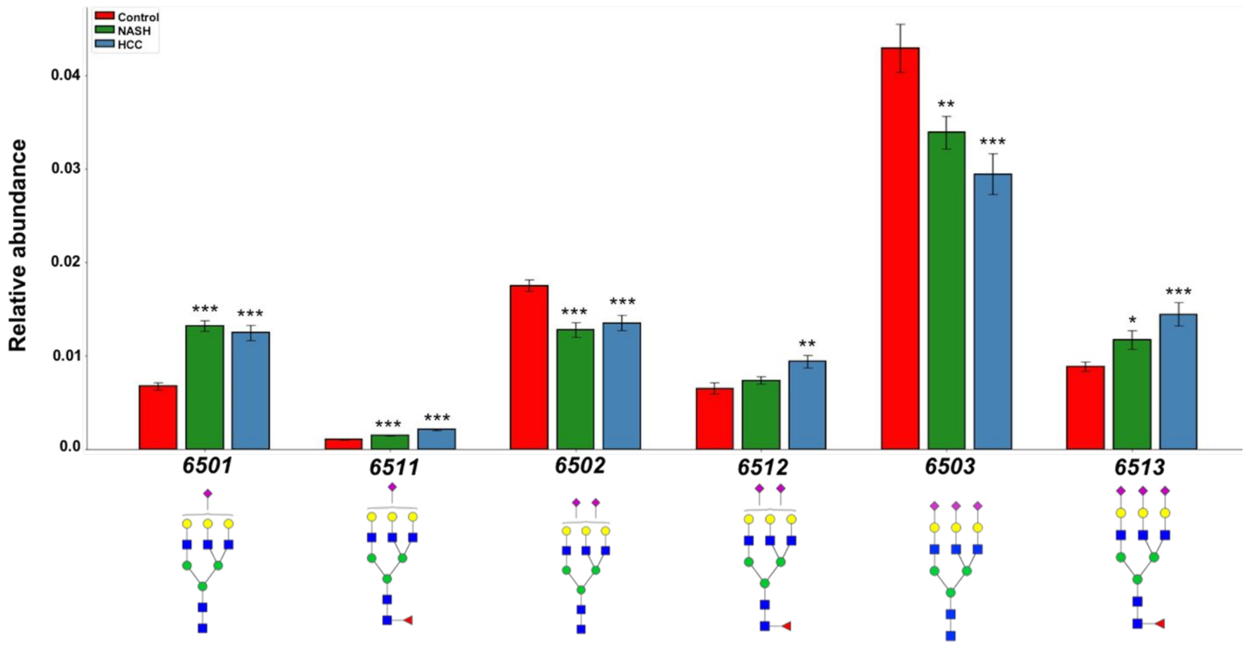
Control
NASH
HCC
Relative abundance
0.04
0.03
0.02
0.01
0.0
6501
6511
6502
6512
6503
6513

Figure S5.

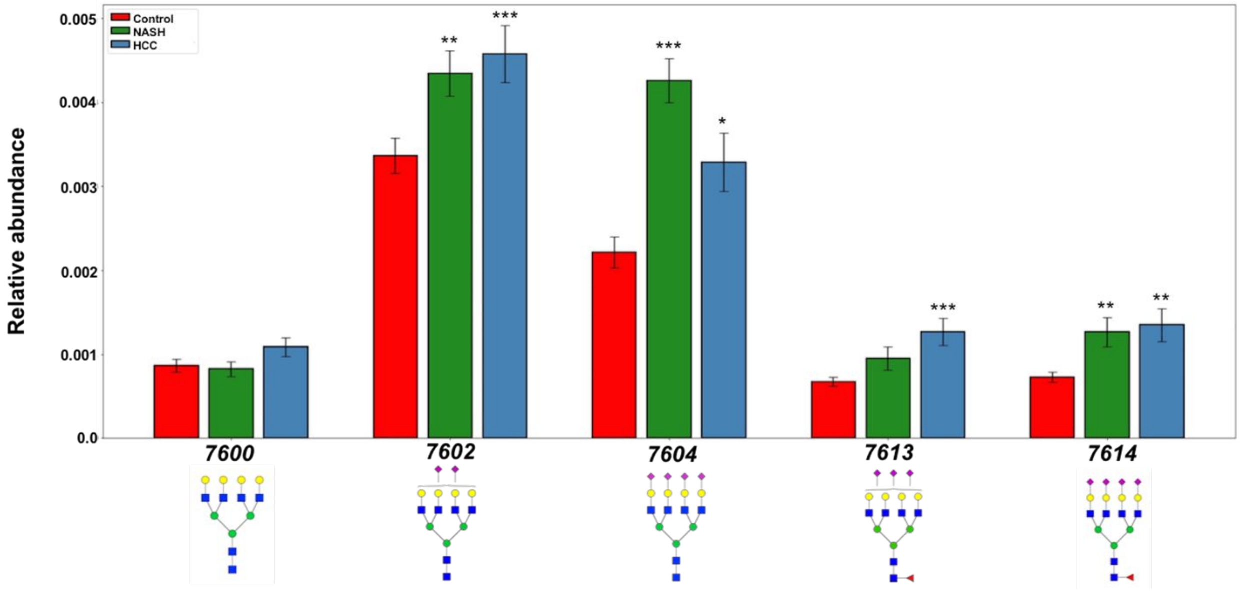
Control
NASH
HCC
Relative abundance
0.005
0.004
0.003
0.002
0.001
0.0
7600
7602
7604
7613
7614

Figure S6.

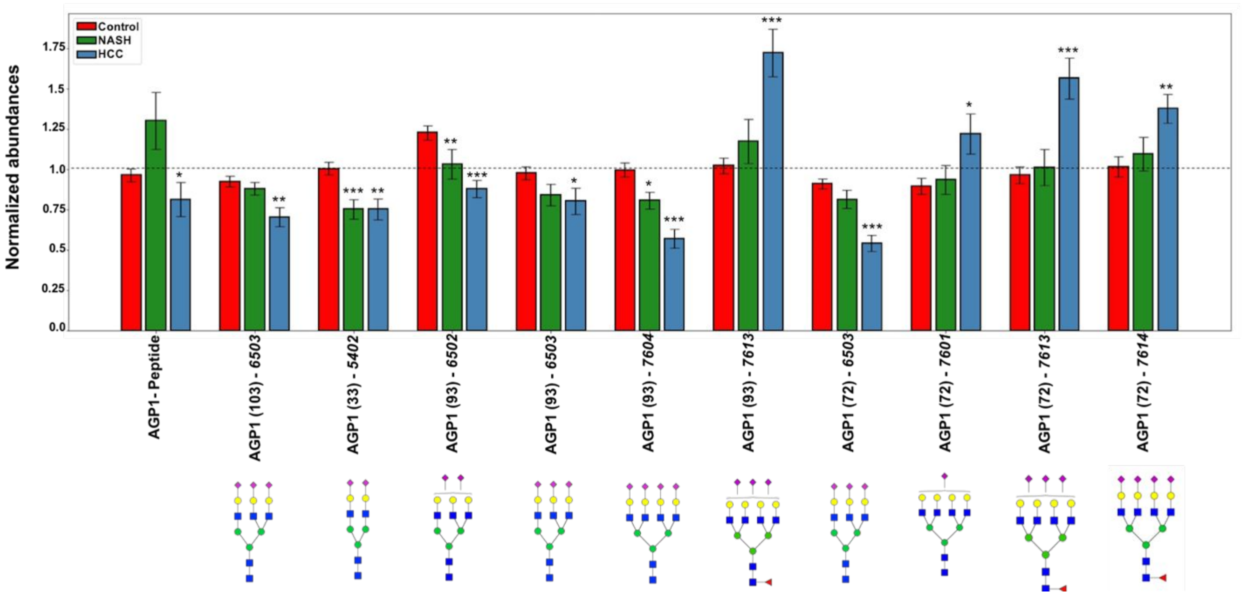

Figure S7.

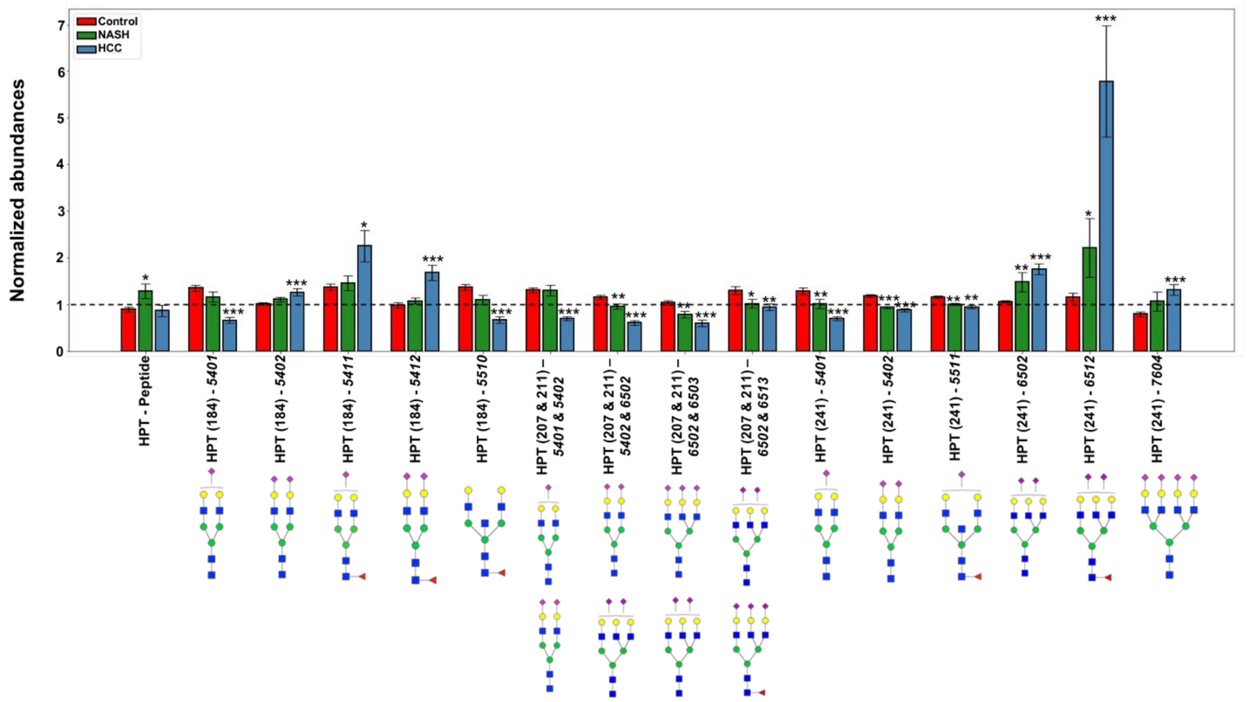
Control
NASH
HCC
Normalized abundances
HPT - Peptide
HPT (184) - 5401
HPT (184) - 5402
HPT (184) - 5411
HPT (184) - 5412
HPT (184) - 5510
HPT (207 & 211) – 5401 & 5402
HPT (207 & 211) – 5402 & 6502
HPT (207 & 211) – 6502 & 6503
HPT (207 & 211) – 6502 & 6513
HPT (241) - 5401
HPT (241) - 5402
HPT (241) - 5511
HPT (241) - 6502
HPT (241) - 6512
HPT (241) - 7604

Figure S8.

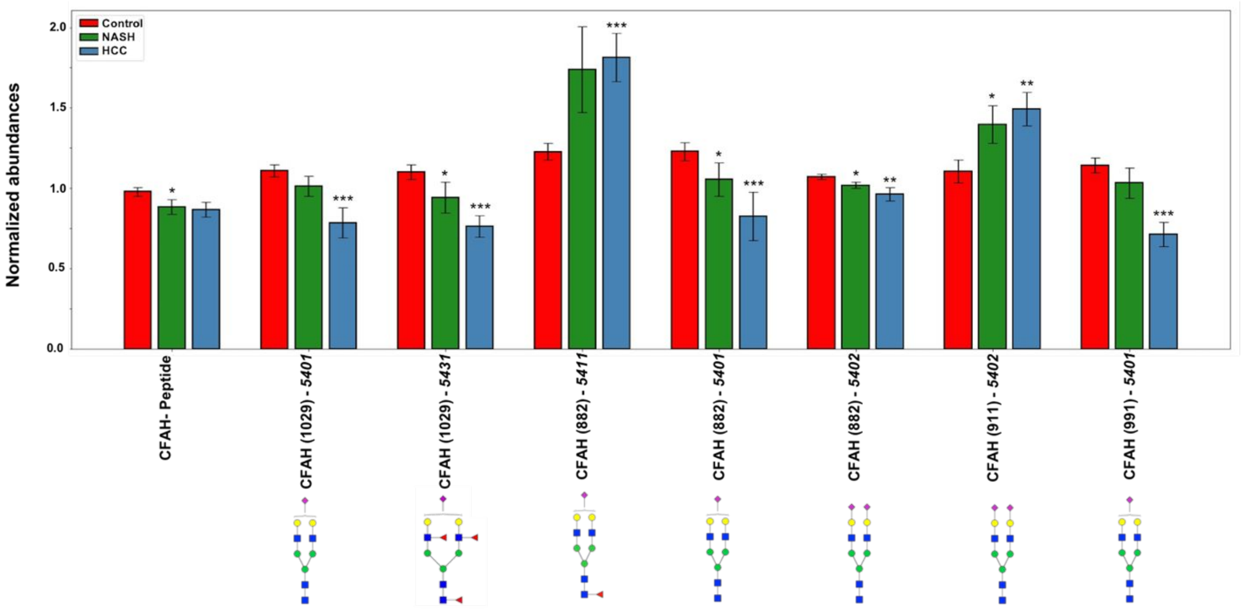
Control
NASH
HCC
Normalized abundances
CFAH- Peptide
CFAH (1029) - 5401
CFAH (1029) - 5431
CFAH (882) - 5411
CFAH (882) - 5401
CFAH (882) - 5402
CFAH (911) - 5402
CFAH (991) - 5401

Figure S9.

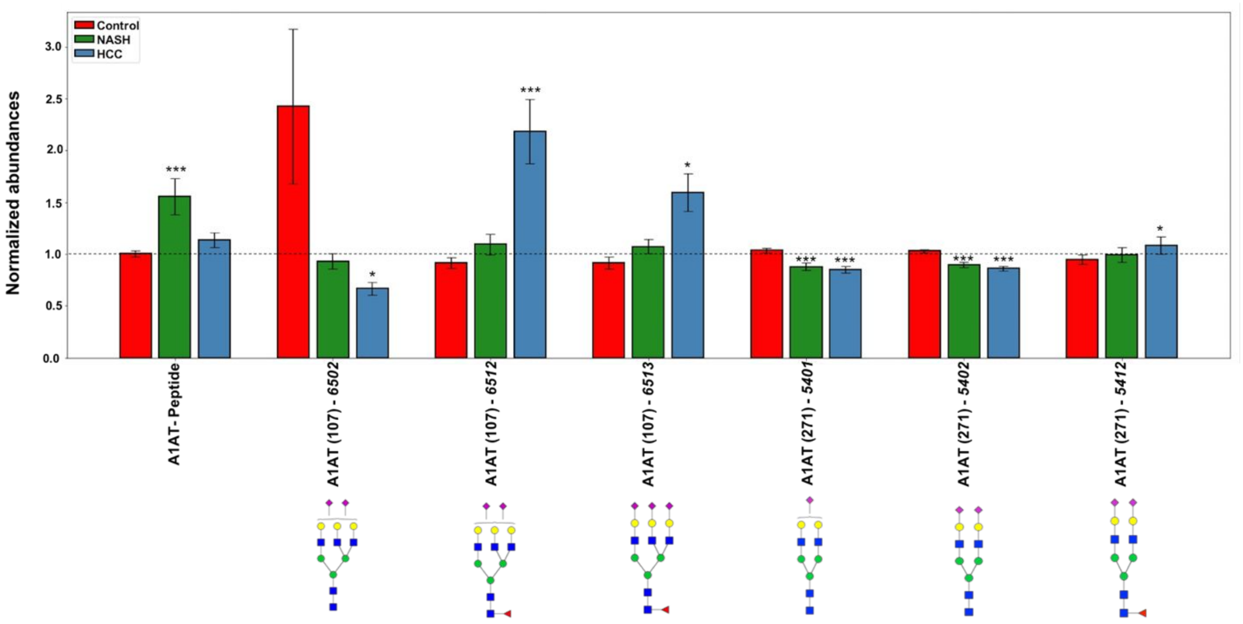
Control
NASH
HCC
Normalized abundances
3.0
2.5
2.0
1.5
1.0
0.5
0.0
***
***
*
***
*
*** ***
*** ***
*
A1AT- Peptide
A1AT (107) - 6502
A1AT (107) - 6512
A1AT (107) - 6513
A1AT (271) - 5401
A1AT (271) - 5402
A1AT (271) - 5412

Figure S10.

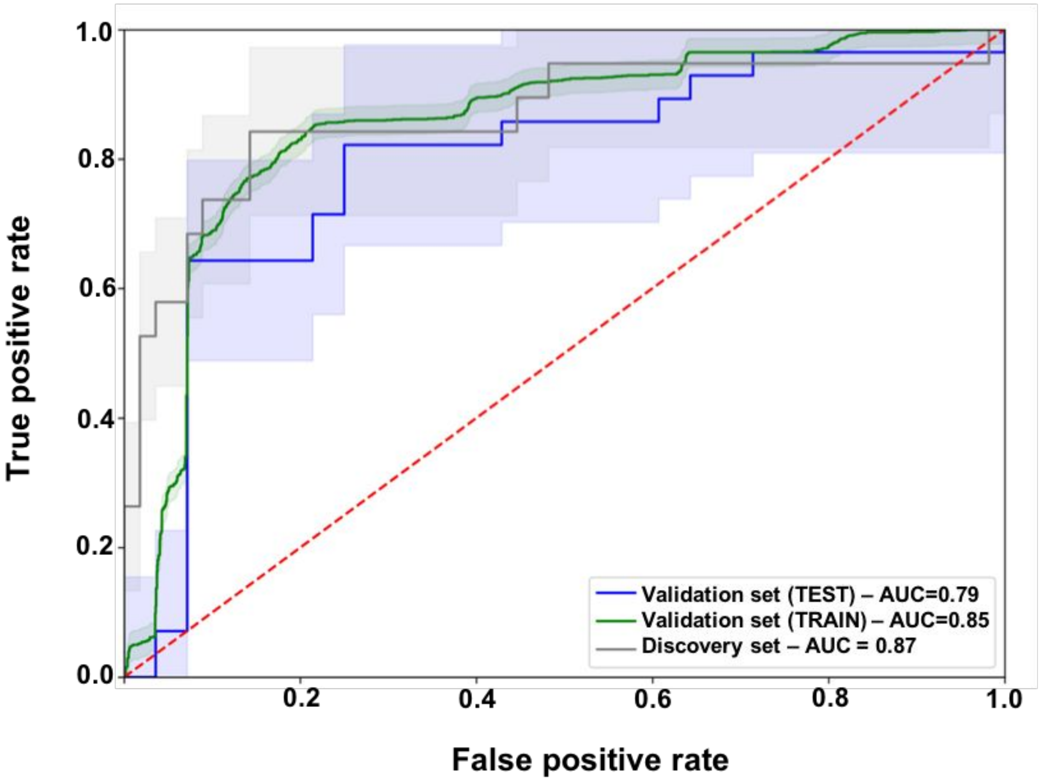

	Accuracy	Specificity	Sensitivity
Validation set (Train)	0.773	0.861	0.685
Validation set (Test)	0.714	0.857	0.571
Discovery set	0.813	0.804	0.842

Figure S11.

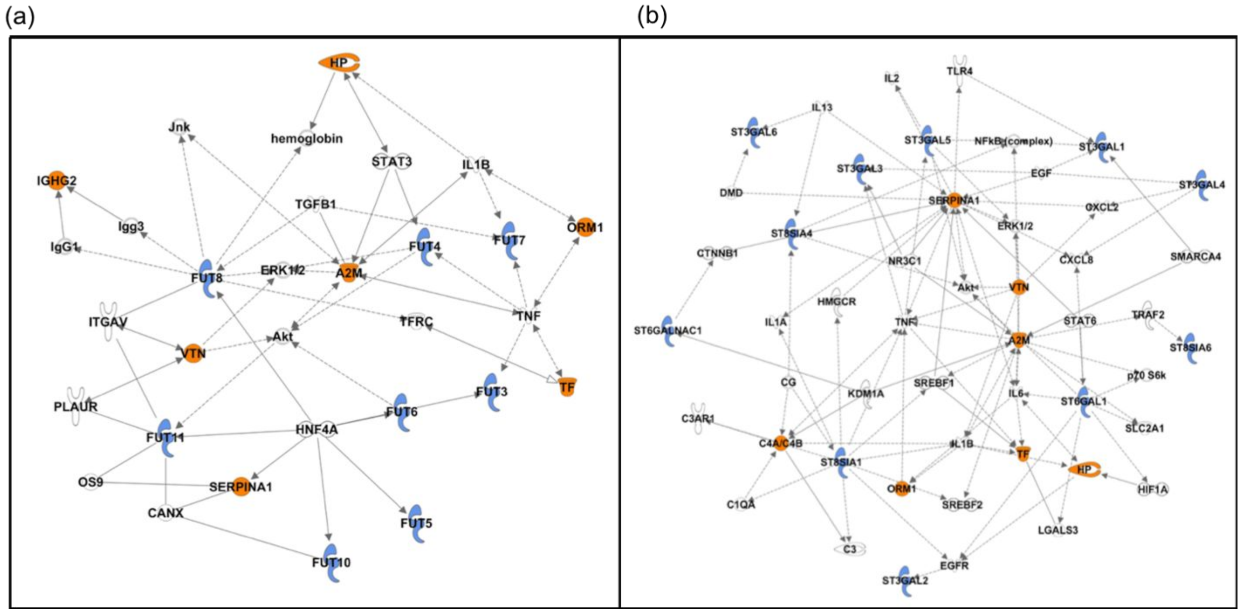
(a)
HP
Jnk
hemoglobin
STAT3
IL1B
IGHG2
TGFB1
Igg3
FUT7
ORM1
IgG1
FUT4
FUT8
ERK1/2
A2M
ITGAV
TNF
TFRC
VTN
Akt
TF
FUT3
PLAUR
FUT6
FUT11
HNF4A
OS9
SERPINA1
CANX
FUT5
FUT10
(b)
TLR4
IL2
IL13
ST3GAL6
ST3GAL5
NFkB (complex)
ST3GAL1
ST3GAL3
EGF
ST3GAL4
DMD
SERPINA1
CXCL2
ST8SIA4
ERK1/2
CTNNB1
NR3C1
CXCL8
SMARCA4
Akt
VTN
HMGCR
IL1A
TNF
STAT6
TRAF2
ST6GALNAC1
A2M
ST8SIA6
p70 S6k
CG
SREBF1
KDM1A
IL6
ST6GAL1
C3AR1
SLC2A1
C4A/C4B
IL1B
TF
ST8SIA1
HP
ORM1
SREBF2
HIF1A
C1QA
LGALS3
C3
EGFR
ST3GAL2

Table S1.

	Number of females	Number of males
Fibrosis stage		
1B	13	10
Steatosis		
2	10	7
3	3	3
Lobular inflammation		
2	9	7
3	4	3
Ballooning		
1	1	
2	4	6
3	8	4

Table S2.

	Number of females	Number of males
Tumor size (cm)		
< 5 cm	1	5
>5 cm	2	9
missing	1	2
T_classification		
T1	1	6
T2	2	6
T3	1	4
N_classification		
N0	3	15
N1	1	1
M_classification		
M0	4	16
Stage	4	6

1		6
II	2	6
III	1	3
IV	1	1
**Grading**		
G1	1	2
G2	1	6
G3	2	5
G4		1
missing		2

Table S3.

Marker	protein	gene	control/ NASH (multiplicative difference)	control/ NASH (p-value)	control/ NASH (FDR)	control/ HCC (multiplicative difference)	control/ HCC (p-value)	control/ HCC (FDR)	Logistic regression model coefficients (NASH vs Rest)	Logistic regression model coefficients (HCC vs Rest)
A1AT (271) – *5401*	A1AT	SERPINA1A	0.85	<0.001	0.005	0.79	<0.001	<0.001	0.013	-0.412
A1AT (271) - *5402*	A1AT	SERPINA1A	0.87	<0.001	<0.001	0.82	<0.001	<0.001	0.099	-0.369
A1BG (179) – *5402*	A1BG	A1BG	1.17	<0.001	0.003	1.28	<0.001	<0.001	0.17	0.056
A2MG (247) – *5402*	A2MG	A2M	1.23	<0.001	0.002	1.47	<0.001	<0.001	-0.045	0.235
A2MG (55) - *5402*	A2MG	A2M	1.17	0.003	0.016	1.34	<0.001	<0.001	-0.108	0.327
A2MG (869) – *6200*	A2MG	A2M	0.87	0.013	0.049	0.68	<0.001	<0.001	0.576	-0.435

AACT (106) - *7604*	AACT	SERPINA3	0.45	<0.001	0.001	0.63	0.003	0.009	-0.287	-0.0507
AGP1 (33) – *5402*	AGP1	ORM1	0.72	<0.001	0.003	0.75	0.002	0.008	-0.138	0.11
AGP1 (93) - *6502*	AGP1	ORM1	0.82	0.009	0.038	0.69	<0.001	<0.001	0.119	-0.701
APOC3 (74) – *1102*	APOC3	APOC3	1.53	<0.001	<0.001	1.77	<0.001	<0.001	0.312	-0.123
APOC3 (74) – *1202*	APOC3	APOC3	1.73	0.001	0.008	2.55	<0.001	<0.001	0.31	0.071
APOC3 (74) – *1300*	APOC3	APOC3	2.01	<0.001	0.005	7.53	<0.001	<0.001	-1.44	0.588
APOC3 (74) – *2110*	APOC3	APOC3	1.93	<0.001	<0.001	2.98	<0.001	<0.001	0.55	0.391
APOM (135) – *5421*	APOM	APOM	1.62	0.014	0.049	2.3	<0.001	0.002	-0.14	0.624
CFAI (70) – *5401*	CFAI	CFI	0.76	0.004	0.024	0.74	0.004	0.0124	-0.68	0.075
CLUS (374) – *6501*	CLUS	CLU	1.46	<0.001	0.005	1.53	<0.001	0.003	1.119	-0.644
CO4A (1328) – *5402*	CO4A	C4A	1.17	<0.001	<0.001	1.39	<0.001	<0.001	0.398	0.45
CO6 (324) – *5200*	CO6	C6	1.57	0.005	0.025	2.42	<0.001	<0.001	-0.295	0.082
CO6 (324) – *5400*	CO6	C6	1.76	0.002	0.01	1.9	0.008	0.023	0.186	0.101
CO8A (437) - *5200*	CO8A	C8A	0.72	0.011	0.043	0.57	<0.001	<0.001	-0.277	-0.974

CO8A (437) – *5410*	CO8A	C8A	1.43	0.008	0.035	1.75	0.002	0.006	0.274	-0.122
HPT (207 & 211) – *5401& 6502*	HPT	HP	0.82	0.006	0.031	0.54	<0.001	<0.001	0.769	-1.036
HPT (207 & 211) – *6502 & 6503*	HPT	HP	0.72	0.005	0.025	0.56	<0.001	<0.001	0.335	-0.606
HPT (241) – *5401*	HPT	HP	0.76	0.007	0.033	0.55	<0.001	<0.001	0.104	-0.743
HPT (241) – *5402*	HPT	HP	0.8	<0.001	<0.001	0.75	<0.001	<0.001	-0.907	-0.131
HPT (241) – *5511*	HPT	HP	0.88	0.005	0.027	0.85	0.002	0.008	-0.318	-0.249
HPT (241) – *6502*	HPT	HP	1.25	0.004	0.021	1.68	<0.001	<0.001	-0.417	0.508
IGA2 (205) – 5510	IGA2	IGHA2	0.42	0.003	0.016	0.08	<0.001	<0.001	-0.592	-0.558
IGG2 (297) – *4400*	IGG2	IGHG2	0.69	0.004	0.022	0.52	<0.001	<0.001	-0.906	-0.847
IGG2 (297) – *4411*	IGG2	IGHG2	1.24	0.008	0.035	1.59	<0.001	<0.001	0.371	0.181
IGM (209) – *5401*	IGM	IGHM	1.5	0.003	0.016	1.47	0.011	0.031	0.17	0.266
KLKB1 (494) – *5401*	KLKB1	KLKB1	1.7	<0.001	0.001	2.87	<0.001	<0.001	0.017	0.086

KLKB1 (494) – *5402*	KLKB1	KLKB1	1.84	0.003	0.017	2.99	<0.001	<0.001	0.223	-0.331
KLKB1 (494) – *5410*	KLKB1	KLKB1	1.27	0.01	0.041	1.79	<0.001	<0.001	-0.037	0.829
KLKB1 (494) – *6503*	KLKB1	KLKB1	1.51	<0.001	0.002	1.6	<0.001	<0.001	0.707	-0.646
TRFE (432) – *5402*	TRFE	TF	1.19	0.001	0.008	1.66	<0.001	<0.001	-0.938	1.004
TRFE (432) – *6501*	TRFE	TF	1.24	<0.001	0.001	1.47	<0.001	<0.001	0.204	0.146
TRFE (432) – *6502*	TRFE	TF	1.13	0.013	0.049	1.42	<0.001	<0.001	-0.539	0.2
VTNC (169) – *5401*	VTNC	VTN	0.71	<0.001	0.002	0.54	<0.001	<0.001	-0.28	-0.238
ZA2G (112) - *5402*	ZA2G	AZGP1	1.49	0.008	0.034	2.06	<0.001	<0.001	-0.308	0.171

Table S4.

Marker	control/NASH (multiplicative difference)	control/ NASH (p-value)	control/ NASH (FDR)	control/HCC (multiplicative difference)	control/HCC (p-value)	control/HCC (FDR)
A1AT_peptide	1.39	<0.001	0.001	1.12	0.1	0.176
A1AT (107) - *6502*	0.76	0.169	0.304	0.6	0.029	0.064
A1AT (107) - *6512*	1.17	0.105	0.219	2.03	<0.001	<0.001
A1AT (107) - *6513*	1.24	0.066	0.157	1.58	0.018	0.043
A1AT (271) - *5401*	0.85	<0.001	0.005	0.79	<0.001	<0.001
A1AT (271) - *5402*	0.87	<0.001	<0.001	0.82	<0.001	<0.001
A1AT (271) - *5412*	0.55	<0.001	<0.001	0.71	<0.001	<0.001

A2MG_peptide	0.85	0.053	0.138	1.26	0.029	0.064
A2MG (1424) - *5401*	0.97	0.779	0.841	0.82	0.043	0.088
A2MG (1424) - *5402*	1.07	0.385	0.542	1.58	<0.001	<0.001
A2MG (247) - *5200*	0.91	0.26	0.411	0.65	<0.001	<0.001
A2MG (247) - *5402*	1.24	<0.001	0.002	1.47	<0.001	<0.001
A2MG (55) - *5402*	1.17	0.003	0.016	1.34	<0.001	<0.001
A2MG (55) - *5411*	0.96	0.586	0.712	0.69	<0.001	<0.001
A2MG (55) - *5412*	0.94	0.391	0.545	0.67	<0.001	<0.001
A2MG (869) - *5200*	0.95	0.313	0.474	0.74	<0.001	<0.001
A2MG (869) - *5401*	1.05	0.366	0.531	1.13	0.045	0.092
A2MG (869) - *5402*	1.13	0.023	0.071	1.17	0.015	0.037
A2MG (869) - *6200*	0.87	0.013	0.049	0.68	<0.001	<0.001
A2MG (869) - 6300	0.91	0.239	0.391	0.62	<0.001	<0.001
AGP1_peptide	1.13	0.22	0.367	0.8	0.019	0.046
AGP1 (103) - *6503*	0.97	0.6	0.722	0.77	0.002	0.008
AGP1 (33) - *5402*	0.72	<0.001	0.003	0.75	0.002	0.008
AGP1 (93) - 6502	0.82	0.009	0.038	0.69	<0.001	<0.001
AGP1 (93) - *6503*	0.85	0.057	0.145	0.77	0.019	0.044
AGP1 (93) - *7613*	1.03	0.776	0.841	1.63	<0.001	<0.001
AGP1 (93) - *7604*	0.83	0.022	0.069	0.5	<0.001	<0.001
AGP12 (72) - *6503*	0.87	0.051	0.136	0.59	<0.001	<0.001
AGP12 (72) - *7601*	1.01	0.908	0.932	1.33	0.016	0.04
AGP12 (72) - *7613*	1.01	0.943	0.955	1.55	<0.001	0.003
AGP12 (72) - *7614*	1.12	0.373	0.545	1.43	0.009	0.024
CFAH_peptide	0.89	0.028	0.084	0.9	0.067	0.127
CFAH (1029) - *5401*	0.91	0.201	0.341	0.67	<0.001	<0.001
CFAH (1029) - *5431*	0.79	0.023	0.0708	0.65	<0.001	<0.001
CFAH (882) - *5411*	1.2	0.095	0.203	1.49	<0.001	<0.001
CFAH (882) - *5401*	0.83	0.058	0.146	0.56	<0.001	<0.001
CFAH (882) - *5402*	0.95	0.048	0.129	0.88	0.006	0.017
CFAH (911) - *5402*	1.29	0.015	0.053	1.35	0.009	0.024
CFAH (911) - *5401*	0.86	0.082	0.183	0.61	<0.001	<0.001

HPT_peptide	1.35	0.015	0.053	0.85	0.315	0.423
HPT (184) - *5401*	0.834	0.162	0.295	0.48	<0.001	<0.001
HPT (184) - *5402*	1.09	0.061	0.151	1.26	<0.001	<0.001
HPT (184) - *5411*	1.03	0.776	0.841	1.36	0.027	0.063
HPT (184) - *5412*	1.14	0.176	0.312	1.78	<0.001	<0.001
HPT (184) - *5510*	0.78	0.062	0.1501	0.47	<0.001	<0.001
HPT (207 & 211) – *5401* & *5402*	0.96	0.642	0.756	0.55	<0.001	<0.001
HPT (207 & 211) – *5402* & *6502*	0.82	0.006	0.031	0.54	<0.001	<0.001
HPT (207 & 211) – *6502* & *6503*	0.72	0.005	0.025	0.56	<0.001	<0.001
HPT (207 & 211) – *6502* & *6513*	0.67	0.041	0.116	0.7	0.009	0.025
HPT (241) - *5401*	0.76	0.007	0.033	0.55	<0.001	<0.001
HPT (241) - *5402*	0.8	<0.001	<0.001	0.75	<0.001	<0.001
HPT (241) - *5511*	0.88	0.005	0.027	0.85	0.002	0.008
HPT (241) - *6502*	1.25	0.004	0.021	1.68	<0.001	<0.001
HPT (241) - *6512*	1.44	0.014	0.051	3.63	<0.001	<0.001
HPT (241) - *7604*	1.08	0.599	0.722	1.74	<0.001	<0.001

Table S5.

Glycoform Code	Glycoform Code (Hex – mannose/galactose, HexNAc – GlcNAc/GalNAc, Fuc – Fucose, NeuAc – Sialylic acid)	Structure

1102	Hex(1) HexNAc(1)Fuc(0)NeuAc(2)	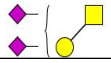
1202	Hex(1)HexNAc(2)Fuc(0)NeuAc(2)	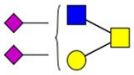
1300	Hex(1)HexNAc(3)Fuc(0)NeuAc(0)	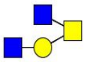
2110	Hex(2)HexNAc(1)Fuc(1)NeuAc(0)	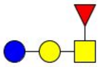
4400	Hex(4)HexNAc(4)Fuc(0)NeuAc(0)	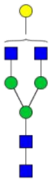
4411	Hex(4)HexNAc(4)Fuc(1)NeuAc(1)	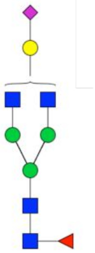
5200	Hex(5)HexNAc(2)Fuc(0)NeuAc(0)	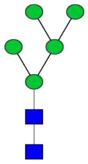

5400	Hex(5)HexNAc(4)Fuc(0)NeuAc(0)	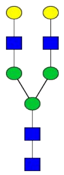
5401	Hex(5)HexNAc(4)Fuc(0)NeuAc(1)	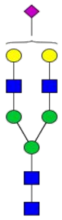
5402	Hex(5)HexNAc(4)Fuc(0)NeuAc(2)	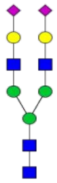
5410	Hex(5)HexNAc(4)Fuc(1)NeuAc(0)	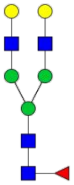
5411	Hex(5)HexNAc(4)Fuc(1)NeuAc(1)	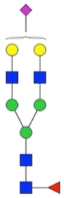

5412	Hex(5)HexNAc(4)Fuc(1)NeuAc(2)	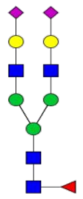
5421	Hex(5)HexNAc(4)Fuc(2)NeuAc(1)	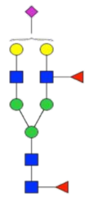
5431	Hex(5)HexNAc(4)Fuc(3)NeuAc(1)	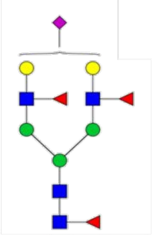
5510	Hex(5)HexNAc(5)Fuc(1)NeuAc(0)	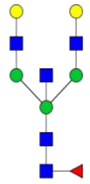
5511	Hex(5)HexNAc(5)Fuc(1)NeuAc(1)	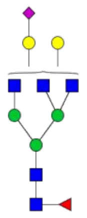

6200	Hex(6)HexNAc(2)Fuc(0)NeuAc(0)	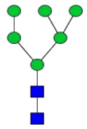
6300	Hex(6)HexNAc(3)Fuc(0)NeuAc(0)	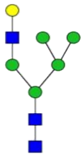
6500	Hex(6)HexNAc(5)Fuc(0)NeuAc(0)	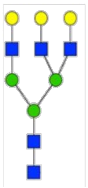
6501	Hex(6)HexNAc(5)Fuc(0)NeuAc(1)	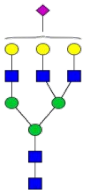
6502	Hex(6)HexNAc(5)Fuc(0)NeuAc(2)	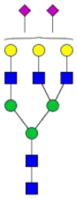

6503	Hex(6)HexNAc(5)Fuc(0)NeuAc(3)	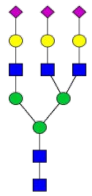
6511	Hex(6)HexNAc(5)Fuc(1)NeuAc(1)	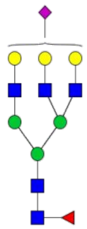
6512	Hex(6)HexNAc(5)Fuc(1)NeuAc(2)	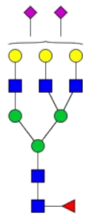
6513	Hex(6)HexNAc(5)Fuc(1)NeuAc(3)	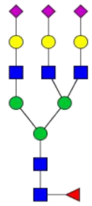
7600	Hex(7)HexNAc(6)Fuc(0)NeuAc(0)	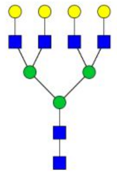

7601	Hex(7)HexNAc(6)Fuc(0)NeuAc(1)	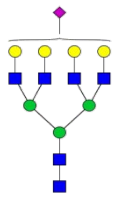
7601	Hex(7)HexNAc(6)Fuc(0)NeuAc(2)	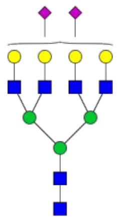
7604	Hex(7)HexNAc(6)Fuc(0)NeuAc(4)	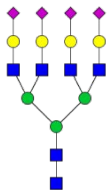
7613	Hex(7)HexNAc(6)Fuc(1)NeuAc(3)	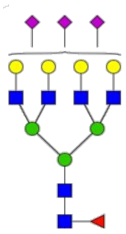

7614	Hex(7)HexNAc(6)Fuc(1)NeuAc(4)	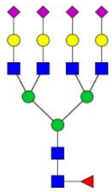

Table S6.

Upstream regulator	Molecule type	p-value of overlap	Target molecules in dataset
**SREBF1**	transcription regulator	7.22E-08	APOC3, CFAI, IGM, A1AT, AACT, TRFE
**IL6**	cytokine	2.15E-07	A2MG, CLUS, HPT, IGM, AGP1, A1AT, AACT, TRFE
**FOXA2**	transcription regulator	1.94E-06	A2MG, APOC3, APOM, A1AT, TRFE
**HNF1A**	transcription regulator	3.18E-06	APOC3, APOM, CO8A, CFAI, A1AT, VTNC
**HNF4A**	transcription regulator	5.26E-06	A1BG, APOC3, APOM, CO4A/CO4B, CO6, AGP1, A1AT, AACT, TRFE, VTNC
**IL6ST**	transmembrane receptor	2.32E-05	A2MG, HPT, AGP1
**PPARA**	ligand-dependent nuclear receptor	4.91E-05	APOC3, APOM, CO6, CO8A, CFAI
**IL6R**	transmembrane receptor	5.91E-05	A2MG, HPT, AACT
**JUN**	transcription regulator	6.27E-05	A2MG, APOC3, APOM, CLUS, TRFE
**IL1A**	cytokine	6.34E-05	CO4A/CO4B, AGP1, A1AT, AACT
